# Post-graduate medical education in the time of COVID-19: Not a remotely simple task

**DOI:** 10.3389/fendo.2022.980505

**Published:** 2022-09-16

**Authors:** Giulio R. Romeo, Yousaf A. Shaikh, Roeland J. W. Middelbeek

**Affiliations:** ^1^ Joslin Diabetes Center, One Joslin Pl, Boston, MA, United States; ^2^ Department of Medicine, Beth Israel Deaconess Medical Center, Boston, MA, United States; ^3^ Harvard Medical School, Boston, MA, United States; ^4^ Department of Medicine, UMass Memorial Medical Center, Worcester, MA, United States

**Keywords:** graduate medical education, telemedicine, diabetes, HbA1c, CGM – continuous glucose monitoring, fellowship, type 1 diabetes, care delivery innovation

## Abstract

The COVID-19 pandemic has stimulated a rapid shift towards telemedicine, which has had tremendous repercussions on all domains of the healthcare ecosystem. The effects of the transition to telemedicine on post-graduate medical education and on patient care provided by trainees have not been fully elucidated. Focusing on the multifaceted scope of endocrinology teaching clinics, the experience garnered by endocrinology fellows, preceptors, and patients through the adoption of virtual visits has shed new light on relevant challenges that require specific attention. First, we identified a divergent trend in glycated hemoglobin in people with type 1 diabetes according to their use of continuous glucose monitoring (CGM). Second, the patient’s perspective highlighted positive aspects, including expanded options for clinical care, but also limitations in communication with clinicians for people without access to videoconferencing tools or EHR-based portals. Finally, regarding medical training evaluation and skills-based learning, academic centers, professional organizations, and clinical educators should develop new teaching curricula suitable for a telemedicine-based environment. While simultaneously facing numerous pressures, fellows can potentially spearhead new models of care delivery and innovative approaches to clinical education leveraging telemedicine.

## Introduction

Bedside teaching based on the in-person interaction between a teacher, a learner, and the patient has been a cornerstone of medical education even before the inception of medical schools (1). Examples of the experiential model of medical training can be dated back to ancient Greece where healers would be joined by aspiring students to treat patients in temples called Asclepia (named after *Asklepios*, the god of medicine). Indeed, it is believed that Hippocrates (*ca*. 460 BC- *ca*. 370 BC) practiced in the Asclepion of Kos where he, and presumably some of his ‘apprentices’ (2), compiled the Hippocratic Corpus (3), which influenced the style of medical teaching for centuries, and noted that “not only must the physician show himself prepared to do what is necessary, he must also secure the co-operation of the patient, the attendants, and of external circumstances” (4).

The impact of ‘external circumstances’ on medical training and patient care has never been more conspicuous than during the ongoing coronavirus disease 2019 (COVID-19) pandemic. Forced by the need of social distancing, medical schools and post-graduate training programs promptly pivoted toward remote learning and patient care as replacement of the traditional clinical teaching sites. Albeit the implementation of these swift changes generally should be seen as a testament to the creativity of teaching institutions and the resilience of the US healthcare system, many questions remain unanswered regarding the short- and long-term consequences of this disruption on medical training as well as clinical care in the teaching setting. As outlined by the Accreditation Council for Graduate Medical Education (ACGME), the assessment of trainee’s progression and readiness for independent practice is predicated on the achievement of six core competencies (Patient Care, Medical Knowledge, Professionalism, Interpersonal and Communication Skills, Practice-based Learning and Improvement, and Systems-based Practice) that are closely linked to case-volume exposure over a prespecified time (e.g. one to three years for the majority of internal medicine subspecialty fellowships). In this context, the rapid transition from in-person to remote patient care and teaching due to COVID-19, and the deployment of residents and fellows to COVID-dedicated services outside their specialty boundaries, have been challenging the learning experience of all trainees and, possibly, their ability to reach key milestones in their chosen specialty.

Here, we will discuss the implications of the switch to telemedicine on *both* the curriculum of endocrinology fellows and the care delivered within diabetes-focused fellows’ clinics. Specifically, we set out to address three questions: 1) How does access to remote digital monitoring affect outcomes for people with type 1 diabetes followed by endocrinology trainees; 2) What are the benefits and obstacles produced by remote teaching within longitudinal outpatient fellows’ clinics?; 3) How should the ‘conventional’ educational framework be modified to ensure both a productive learning environment and adequate fellows’ assessment when using telemedicine?

## Methods

### Quality assurance/Quality improvement

As reference, the structure of our fellows-centric teaching clinic is built on longitudinal care provided by our eight fellows to their respective patient panels, with the mentorship of ten core preceptors rotating through the clinic. To assess the impact of telemedicine on diabetes care delivered by our fellows, we queried the electronic health record system at the Joslin Diabetes Center (JDC) for non-pregnant adult people with type 1 diabetes (T1D) and diabetes duration >1 year who had been followed in the endocrine fellows teaching clinic at JDC. Each patient was cared for by a single Endocrinology Fellow as part of their own patient panel, and precepted by an attending endocrinologist at JDC. Specifically, we determined changes in glycated hemoglobin (HbA1c) in people who had an office visit between November 24, 2019 and March 24, 2020 (4 months prior to the transition to telemedicine; referred to as V1), had at least one remote visit between March 24, 2020 and Sept 14, 2020, when on-site visits were resumed (V2), and at least one office visit within the subsequent 4 months (V3). People who used CGM from V1 through V3 were considered “CGM-users” (*n*=23). All other people, including those who used CGM intermittently, were considered “non-CGM users” (*n*=17). Changes in mean HbA1c both within and between the two groups from V1 to V3 were analyzed using a paired or unpaired Student’s t-test, respectively, with type 1 error of 0.05. Frequencies of nominal variables were compared using Fisher’s exact test. Data were expressed as mean ± SD or mean ± SEM.

### Post-remote visit patients’ survey

As part of a larger QI-initiative, we queried 25 randomly selected persons within two weeks from their remote visit (phone or video) in our diabetes Fellows’ clinic, which serves as a referral center for a demographically diverse group of people with diabetes. Generally, fellows care for patients longitudinally over a 2-3 year period, with no cross-over of panels between fellows. A 10-question survey was administered between June and August 2020 to assess satisfaction level with the virtual visit and retention of therapeutic recommendations. Answers 1-8 were graded on a 5-point Likert scale.

### Fellows’ and preceptors’ survey

A 7-question survey was administered to both endocrinology fellows (n=7) and preceptors (n=10) staffing the diabetes teaching clinic at JDC between June and August 2020. Answers to questions 1-5 were graded on a 5-point Likert scale.

## Discussion

### The patient’s perspective

The COVID-19 pandemic has generated widespread delays in access to both urgent and non-urgent care across all demographics and ethnicities (5), due to disruption of healthcare delivery systems (e.g. cancelled appointments), and patient-specific hurdles such as limited access to technology, and loss of income or employer-sponsored insurance (6, 7). The marked reduction in admissions for acute non-COVID-19 related conditions during the pandemic (8) also suggests that patients may have deferred urgent evaluation, in part due to fear of exposure to COVID-19 in medical facilities (9). With regard to chronic diseases, a national survey found that the frequency of forgoing planned medical care or missing medications was higher in respondents who were unemployed or from lower-income households (10), which are major social determinants for the disproportionately high prevalence of diabetes among non-White US adults (11).

To limit the effects of gaps in medical care during the current crisis, the Coronavirus Aid, Relief, and Economic Security (CARES) Act allowed the expansion of types of services delivered *via* telehealth and supported reimbursement for telemedicine (12). While healthcare organizations worked tirelessly to optimize clinical operations through the rapid shift to telemedicine, the consequences of this transition on the management of complex conditions like diabetes remain to be determined.

Within our specialized referral center, the diabetes clinic staffed by our endocrinology fellows and their preceptors serves a diverse population, including a particularly large group of people with T1D and variable access to diabetes technology. As part of a QA and QI initiative, we assessed the impact of remote digitial monitoring (RDM) during the transition to telemedicine in people with T1D who were exclusively cared for by our fellows.

**Figure 1:**
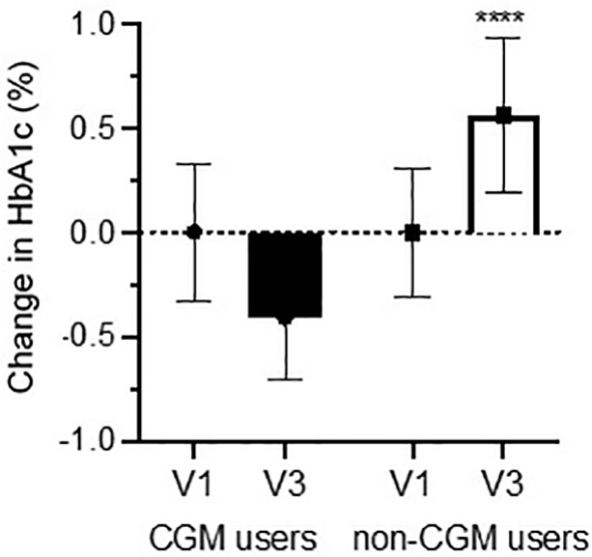
Baseline-corrected change in HbA1c pre- & post-remote visit.. Change in HbA1c for people with T1D who used continuous glucose monitoring (CGM users; *n*=23) or not (non-CGM users, *n*=17) before (V1), during (March-September 2020), and after (V3) the transition to virtual visits. Bars represent the mean change between the value obtained at V1 and V3, for their respective group. All patients were followed exclusively in our longitudinal fellows’ clinic and by a single fellow. Data are expressed as mean ± SEM. ****P<0.0001.

When stratified for the use of continuous glucose monitoring (CGM), baseline mean HbA1c values were similar in CGM users and non-CGM users (8.0 ± 1.1% and 8.1% ± 0.9%, respectively). Remarkably, in CGM users mean HbA1c decreased by 0.4% ± 0.29% in V3, when compared to V1; conversely, non-CGM users demonstrated a mean increase in HbA1c of 0.56% ± 0.37%, with a difference between the means of ~1% (*P*=0.0001 vs CGM users; **Figure 1**). In addition, the change in mean HbA1c from the respective baseline was statistically significant for both the CGM users and non-CGM users (*P*<0.001 for both within-group differences). Age, gender distribution, and diabetes duration were similar between the two groups (**Table 1**). To place the difference in HbA1c in perspective, a 1% HbA1c reduction has been associated with a significant reduction in the risk of developing diabetes-related complications, for both type 1 (13) and type 2 diabetes (14).

**Table 1:** Characteristics of CGM users and non-CGM users.

	CGM users (n=23)	Non-CGM users (n=17)	*P*
Age (years)	43.2 ± 15.1	44.7 ± 15.9	0.76
Gender; M/F (%/%)	9/13 (41/59)	5/12 (29/71)	0.51
DM duration (years)	24.9 ± 14.7	22.1 ± 12.5	0.52
HbA1c (%) at V1	8.0 ± 1.1	8.1 ± 0.9	0.86

Age, gender distribution, diabetes (DM) duration, and baseline HbA1c at visit 1 (V1) were comparable in the continuous glucose monitoring (CGM)-user group and non-CGM users. For continuous variables, data are expressed as mean ± SD. The gender distribution was compared by Fisher’s exact test.

To the best of our knowledge, this is the first observation of differences in HbA1c trend based on CGM use in a teaching clinic, further supporting the essential role of digital technology for diabetes care observed in other settings (15–17). Also, the diverging trajectory of HbA1c between the two groups underscores the need for patient-centered, legislative, and system-based initiatives to facilitate a more widespread adoption of CGM. In this view, the lower use of diabetes technology among minorities, at least for persons with T1D (18, 19), could curtail access to the expanding options of virtual care and further widen ethnic disparities in diabetes outcomes (20). Among other interventions to promote RDM, the reassessment of current criteria for CGM coverage by Medicare and other payers, which are not evidence-based, and the reduction of out-of-pocket cost should be considered logical steps as many patients rely on remote visits, whilst balancing medical care costs and food insecurity.

We applaud new programs established within the American Rescue Plan Act (ARPA) (21) that fund broadband infrastructure projects and provide financial assistance for Internet costs to eligible households; yet, in the short term inequalities in distribution of high-speed connectivity in rural areas and among minorities and the elderly (22) will amplify the shortcomings of the sparse access to diabetes technology in such groups of patients (23).

Collectively, these issues have the potential to affect patient safety, which has not consistently been a top priority through the rapid incorporation of telemedicine into the clinical workflow. Within the scope of our teaching diabetes clinic, we documented challenges related to retention of therapeutic recommendations discussed during virtual visits (especially phone visits) when patients did not have access to ‘open notes’ portals or were not provided with a written summary. Specifically, twelve out of twenty-five patients, randomly selected for a survey within 2 weeks from their remote visit within our teaching clinic, were not able to recall important details of their telehealth visit (e.g. recommended reduction in insulin dosing) that could have resulted in adverse events (e.g. hypoglycemia due to excess insulin administration) (**Table 2**). Patient’s choice of phone rather than video visits, lack of patient portal use, and lack of CGM use were common features in all 12 patients with limited recall of their treatment plan. To address this deficiency, our fellows’ clinic has piloted changes in processes for remote visits and developed a SAF-T pathway (Summarize plan of care and Assess understanding of recommendations during encounter; Forward synopsis of Therapeutic plan after the encounter), which should limit the risk of breakdown in patient communication until much needed, larger initiatives are implemented.

**Table 2:** Survey on remote visit experience for people with diabetes.

	Strongly Disagree	Disagree	Neither Agree nor Disagree	Agree	Strongly Agree
The overall experience of your visit was satisfactory	0	0	2 (8)	15 (60)	8 (32)
The in-training physician addressed my concerns	0	0	1 (4)	12 (48)	12 (48)
The in-training physician was professional and sympathetic	0	0	1 (4)	12 (48)	12 (48)
The senior physician addressed my concerns	0	0	3 (12)	14 (56)	5 (32)
The senior physician was professional and sympathetic	0	0	1 (4)	10 (40)	14 (56)
Prior to the visit, I received clear instructions regarding how to share my glucose monitoring devices (glucometer or continuous glucose monitor) and insulin pump	0	5 (20)	6 (24)	6 (24)	8 (32)
The connection *via* phone (P)/video (V) allowed me to effectively hear and understand my physicians	0	0 P 2 (15) V	1 (8.3) P 1 (7.7) V	6 (50) P 5 (36.4) V	5 (41.6) P 5 (38.4) V
At the end of the visit, my physicians reviewed clearly and in layperson language the changes to my diabetes regimen, if applicable, and the plan for follow-up	0	0	2 (8)	12 (48)	11 (44)
		Yes	No		
After the visit, I was provided with an “electronic” written summary of the plan		12 (48)	13 (52)		
Have you signed up and do you use the Patient Portal?		12 (48)	13 (52)		

Twenty-five people were randomly selected for a survey within two weeks from their remote visit with our teaching clinic. Answers to questions 1-8 are graded on a Likert scale. Data represent the number of responses and percentage of total for each question, n (%).

Telehealth has been a tremendous resource for providing diabetes care through the pandemic and will likely be an integral component of innovative models for the treatment of chronic diseases. On the other hand, the COVID-19 pandemic has unmasked a multifaceted technology gap that threatens to stifle the delivery of care to large groups of patients, with a disproportionate representation of minorities and elderly. As the medical community will be tasked with spearheading new approaches for RDM and digital health, our trainees could be an essential catalyst for solutions that are sustainable, scalable, and promote health benefits for patients across *all* communities.

### Fellows’ training and well-being through the pandemic: A moving target

Among numerous consequences, the COVID-19 pandemic has imposed an unprecedented pressure on the medical education system, and affected the learning experience and the well-being of medical trainees, who have often been deployed to services outside their specialty caring for COVID-19 patients.

#### Clinical fellows’ well-being

Anxiety due to the risk of exposure to the coronavirus, social isolation, and emotional exhaustion – stressors common to all frontline workers (24, 25) – are compounded for many fellows by concerns of lost learning opportunities in their specialty of choice. Addressing repercussions of these factors on the mental health of fellows (and all trainees) should be a priority for any academic center to reduce the risk of ‘burnout’, which had been steadily rising before the pandemic for both practicing and in-training physicians (26).

The risk of burnout is particularly high for non-procedural subspecialties like endocrinology and for female physicians (27), who constitute the majority of the fellows recruited in endocrinology programs (28, 29). The emotional distress and depersonalization characteristic of burnout will likely be exacerbated by the current pandemic, thus threatening the stability of the endocrinology workforce at a moment of great need. In addition, burnout can reduce the engagement of endocrinology clinical educators - including fellows - in teaching activities for students and residents, blunting the impact of the already modest exposure to endocrinology curriculum in medical training. We and others have highlighted that enthusiastic endocrinology fellows are the most effective ‘ambassadors’ of the field and a key conduit to attract top talent into endocrinology programs (30, 31).

How should the ACGME and endocrinology organizations tackle this challenge, specifically pertaining to fellows? How should endocrinology programs support their fellows at this difficult juncture? These questions require a multitiered approach that should extend beyond the current crisis. First, leaders should encourage fellows to share their psychological distress in safe spaces and validate their concerns. The persistent stigma around mental illness in the medical community, both interpersonal and self-stigma, often deters clinicians from seeking help (32). In response to this growing problem, the ACGME emphasized a “culture of well-being” in the 2018 revised Common Program Requirements and its supporting resources (https://acgme.org/What-We-Do/Initiatives/Physician-Well-Being/Resources), and more recently created a “COVID-19 Well-Being Task Force”. Second, healthcare organization should allocate funding to raise awareness about burnout, and facilitate the report (e.g. hotlines) and assistance of clinicians in need. Third, given the recently noted trends among trainees (33), we encourage teaching programs to directly elicit initial signs of burnout with fellows and initiate interventions at multiple levels: a) educational, e.g. design a plan to maintain a productive learning specialty experience through the crisis (see below); b) individual, e.g. address concerns about personal safety and virus transmission to loved ones; c) culture, e.g. promote a climate of partnership among team members.

#### Finding purpose in innovation

With regard to educational interventions, all trainees (and preceptors) adapted to a *modus operandi* for virtual encounters that relies less on physical findings or non-verbal cues. As part of a single-program survey at our institution (N=18, 100% response), 37.5% of fellows and 60% of preceptors felt that the teaching experience during a remote visit was less productive than in-person encounters (**Table 3**). Two frequently noted obstacles were timely access to key patient data (e.g. CGM and insulin pump downloads) and technical challenges of conferencing in all participants, thus leaving lesser time for educational discussions between the fellow and the preceptor. The latter point highlights the importance of defining learning objectives for each virtual visit, allocating sufficient time for teaching (e.g. review of cases and associated literature after the clinic), and developing a more uniform approach to telehealth-based preceptorship.

**Table 3:** Fellows and preceptors survey on remote visits within diabetes-focused teaching clinic.

		Strongly Disagree	Disagree	Neither Agree nor Disagree	Agree	Strongly Agree	
In combination with the (Fellow or Preceptor), you have been able to deliver patient care in an effective and efficient manner for the vast majority of remote encounters.	Fellows	0	0	1 (12.5)	3 (37.5)	4 (50)	
	Preceptors	0	1 (10)	2 (20)	4 (40)	3 (30)	
	Total n (%)	0	1 (5.5)	3 (16.6)	7 (38.8)	7 (38.8)	
In the context of the current workflow of the Fellows’ clinic, Telemedicine allows an adequate amount of time for Fellows’ teaching	Fellows	0	1 (12.5)	0	4 (50)	3 (37.5)	
	Preceptors	0	1 (10)	5 (50)	4 (40)	0	
	Total n (%)	0	2 (11.1)	5 (27.7)	8 (44.4)	3 (16.6)	
In general, you find that the teaching experience of a remote visit is as productive as during an in-person visit	Fellows	0	2 (25)	1 (12.5)	3 (37.5)	2 (25)	
	Preceptors	0	2 (20)	5 (50)	3 (30)	0	
	Total n (%)	0	4 (22.2)	6 (33.3)	6 (33.3)	2 (11.1)	
In general, the connection *via* phone allowed me to effectively hear and understand my patient and the (Fellow or Preceptor)	Fellows	0	1 (12.5)	1 (12.5)	3 (37.5)	3 (37.5)	
	Preceptors	0	0	1 (10)	8 (80)	1 (10)	
	Total n (%)	0	1 (5.5)	2 (11.1)	11 (61.1)	4 (22.2)	
In general, the connection *via* video allowed me to effectively hear and understand my patient and the (Fellow or Preceptor)	Fellows	0	2 (25)	0	2 (25)	4 (50)	
	Preceptors	0	0	2 (22)	5 (55)	2 (22)	
	Total n (%)	0	2 (11.7)	2 (11.7)	7 (41.2)	6 (35.3)	
		Yes		No	Don’t Know	
At the end of the visit, I (Fellow or Preceptor) reviewed in layperson language the changes to diabetes regimen, if applicable, and the plan for follow-up with the (Fellow or Preceptor) and the patient	Fellows	8 (100)		0	0	
	Preceptors	10 (100)		0	0	
	Total	18 (100)		0	0	
After the visit, the patient was provided with a written summary of the plan, either as a mailed hard-copy or in electronic form (that may include information forwarded through the patient portal)	Fellows	3 (37.5)		4 (50)	1 (12.5)	
	Preceptors	2 (20)		1 (10)	7 (70)	
	Total	5 (27.7)		5 (27.7)	8 (44.4)	

Eight fellows and ten preceptors within our teaching clinic were enrolled in a survey within three months from the transition from on-site to remote visits (March-June 2020). Answers to questions 1-5 are graded on a Likert scale. Data represent the number of responses and percentage of total for each question, n (%).

In addition, the engagement of trainees in digital health educational projects can bring additional meaning to their clinical training. In this context, endocrinology fellows should be a driving force in piloting the transformation of traditional, office-based models of diabetes care to novel ways of delivering care to people with DM that leverage RDM. Trainees can provide a unique, less biased perspective to both the design and the identification of barriers in digital health (34, 35). Not only can fellows be insightful ‘superusers’ but they can also coach clinicians who are less familiar with technology-enabled applications. Also, the relatively smaller panel of patients followed by fellows provides the opportunity for more frequent ‘touch points’ and therefore more granular data collection, which are essential features when piloting projects.

### Towards a ‘novel’ educational framework

The COVID-19 pandemic has disrupted many aspects of daily life, including healthcare delivery and medical education. While in-person care has resumed, telemedicine, facilitated by reimbursement changes and in parallel with the development of commercial care delivery platforms, is here to stay. Thus, rethinking post-graduate medical education programs is critical to both ensure a productive learning environment and adequate assessment of fellows’ competencies. Numerous stakeholders are involved, including the ACGME, academic medical centers, and teaching physicians. The six core competencies, as identified by the ACGME, may need to be revised and expanded to incorporate “telemedicine skills”. This is the case for patient care, medical knowledge, practice-based learning & improvement, interpersonal & communication skills, professionalism, and systems-based practice. Communication skills, and patient care appear most directly implicated, but longer-terms effects on other competencies cannot be ignored. As an example, clinical case conferences that occur in hybrid format, may now broaden medical knowledge for a larger audience. Around the time of the clinical encounter, teaching physicians may need to provide feedback in a different manner, which may include reviewing clinic encounters at a dedicated time outside of the (virtual) clinic.

Will there be a role for bedside teaching? Yes, both for procedure-based and office-based medical specialties, in-person (bedside) teaching remains essential. However, all stakeholders in post-graduate medical education should consider developing novel curricula that enhance telemedicine-based teaching for our trainees, as they will deliver patient care in a more diverse and dynamic healthcare ecosystem.

In summary, post-graduate medical training and patient care within teaching clinics has been upended by the COVID pandemic. Not only the transition to telemedicine has underscored the essential role of CGM for T1D care, but also forced the endocrinology medical community to foster training opportunities beyond bedside teaching. It is our hope that endocrinology fellows will head creative solutions to expand telemedicine-based models of diabetes care both in academic centers and underserved communities.

## Data availability statement

The raw data supporting the conclusions of this article will be made available by the authors, without undue reservation.

## Author contributions

GR and RM acquired, analyzed, and interpreted the data, and wrote the manuscript; YS wrote the manuscript. All authors agreed to be accountable for all aspects of the work.

## Acknowledgments

We thank Dr. R.A. Gabbay at the American Diabetes Association for reading our manuscript and providing valuable feedback. We are grateful to all trainees, preceptors, and patients who participated in the surveys.

## Conflict of interest

RM has received research funding from Novo Nordisk, unrelated to this work.

The remaining author declares that the research was conducted in the absence of any commercial or financial relationships that could be construed as a potential conflict of interest.

## Publisher’s note

All claims expressed in this article are solely those of the authors and do not necessarily represent those of their affiliated organizations, or those of the publisher, the editors and the reviewers. Any product that may be evaluated in this article, or claim that may be made by its manufacturer, is not guaranteed or endorsed by the publisher.
